# Absence of *ABL1* exon 2-encoded SH3 residues in *BCR::ABL1* destabilizes the autoinhibited kinase conformation and confers resistance to asciminib

**DOI:** 10.1038/s41375-024-02353-0

**Published:** 2024-07-31

**Authors:** Ariel Leyte-Vidal, RosaAnna DeFilippis, Ian R. Outhwaite, Isabelle Kwan, Ji Young Lee, Carlyn Leavitt, Kaeli B. Miller, Delphine Rea, Aziz M. Rangwala, Kevin Lou, Suhana Patel, Ailin Alvarez, Kevan M. Shokat, Ivet Bahar, Markus A. Seeliger, Neil P. Shah

**Affiliations:** 1 Division of Hematology/Oncology, Department of Medicine, University of California, San Francisco, CA 94143 USA; 2 Department of Biochemistry and Molecular Biology, University of Miami Miller School of Medicine, Miami, FL 33101 USA; 3 Department of Pharmacological Sciences, Renaissance School of Medicine, Stony Brook University, Stony Brook, NY 11794 USA; 4 Laufer Center for Physical and Quantitative Biology, Stony Brook University, Stony Brook, NY 11794 USA; 5 Department of Biochemistry and Cell Biology, Renaissance School of Medicine, Stony Brook University, Stony Brook, NY 11794 USA; 6 Adult Hematology Department, Hôpital Saint-Louis, Paris, France; 7 Department of Cellular and Molecular Pharmacology, University of California, San Francisco, CA 94158 USA; 8 Howard Hughes Medical Institute, University of California, San Francisco, CA 94158 USA; 9 Department of Chemistry, University of California, Berkeley, CA 94720 USA

**Keywords:** Targeted therapies, Translational research

## To the Editor:

**Fig. 1:**
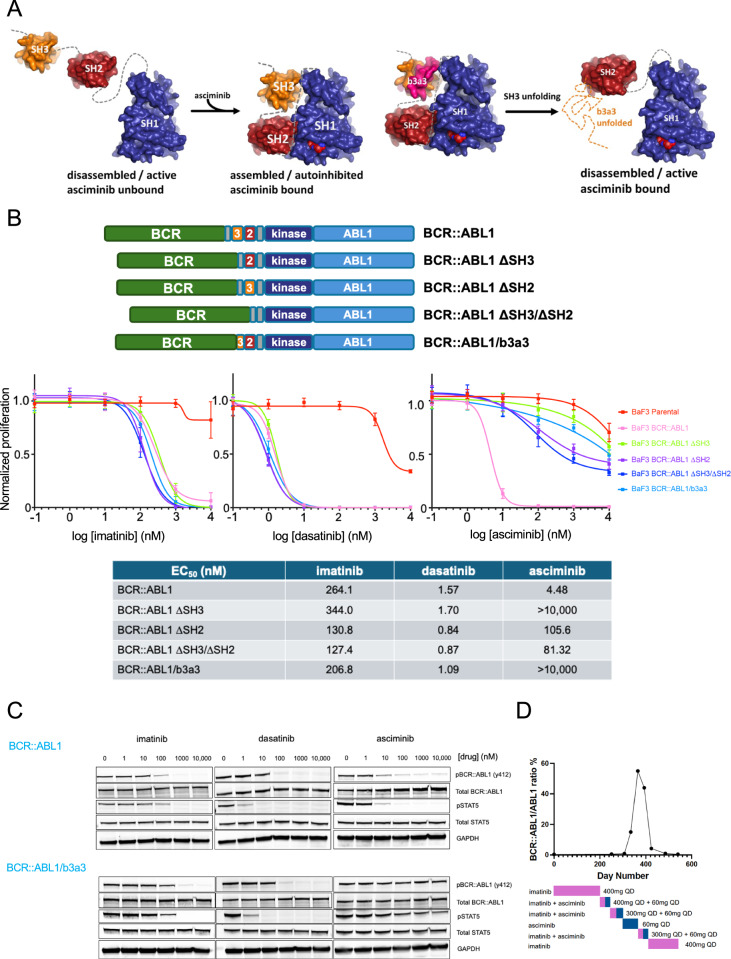
Deletion of SH3, SH2, SH3/SH2 and ABL1 exon2 (resulting in the BCR::ABL1/b3a3 isoform) in BCR::ABL1 confers asciminib resistance. **A** (Left) Schematic depiction of ABL1-SH3 (gold), SH2 (maroon) and SH1 (kinase; blue) domains in an active, disassembled conformation and in an assembled autoinhibited conformation upon binding of asciminib (red). *(Right)* Schematic depiction of sequences encoded by exon 2 [*pink; (“b3a3”)]* in the SH3 domain and hypothesized resultant disruption of autoinhibited conformation despite asciminib binding. **B** (Upper) Schematic representation of BCR::ABL1 deletions. Location of SH3 (“3”), SH2 (“2”) domains is depicted. (Lower) Proliferation assays of pools of Ba/F3 cells transformed to IL-3 independence by BCR::ABL1 isoforms in varying concentrations of TKIs for 48 h and assessed by CellTiter-Glo. Ba/F3 parental cells were grown in the presence of IL-3. Results were performed in technical and biological triplicate. Mean values and standard errors are depicted. Table provides calculated EC_50_ values. **C** Western immunoblot analysis of lysates of Ba/F3 cells transformed by BCR::ABL1 or BCR::ABL1/b3a3 and exposed to TKIs at the concentrations indicated for two hours. **D** Molecular response of a CML patient with BCR::ABL1/b2a3 while on treatment with imatinib and asciminib.

The recently approved BCR::ABL1 tyrosine kinase inhibitor (TKI) asciminib has demonstrated considerable activity and tolerability in newly diagnosed chronic phase chronic myeloid leukemia (CML) patients [1]. In contrast to previously approved BCR::ABL1 TKIs that target the ATP-binding (“orthosteric”) site, asciminib is a first-in-class allosteric TKI that targets the myristoyl-binding pocket in the C-lobe of ABL1 kinase (SRC homology-1; SH1). Asciminib binding induces a closed, inactive kinase conformation that recapitulates physiologic autoinhibition of ABL1 kinase, whereby the SH3 and SH2 domains bind to the kinase domain [2] (Fig. 1A). In addition to point mutations surrounding the myristoyl-binding pocket in the kinase C-lobe that confer clinical resistance to asciminib [3, 4], we recently demonstrated that mutations near the top of the kinase N-lobe unexpectedly confer clinical and/or in vitro asciminib resistance. BCR::ABL1/M244V retains the ability to bind asciminib, implicating disruption of the allosteric mechanism of action as the basis for its resistance to asciminib [5].

BCR::ABL1 variants lacking ABL1 exon 2 occur in a minority of CML patients as a consequence of the chromosome 9 breakpoint occurring 3’ of this exon [6–9], and are referred to as BCR::ABL1/b2a3 or BCR::ABL1/b3a3 depending upon the presence or absence of BCR exon 14. Recent clinical trials with BCR::ABL1 TKIs have largely employed molecular endpoints. However, *BCR::ABL1* transcript levels are only standardized for full-length *BCR::ABL1* isoforms that contain ABL1 exon 2 (“b2a2”, “b3a2”). CML patients with BCR::ABL1/b2a3 or BCR::ABL1/b3a3 variants have been excluded from these studies, although post-marketing experience has demonstrated excellent outcomes with orthosteric TKIs [10–12]. Given the allosteric mechanism of action of asciminib, we sought to formally assess the roles of the SH3 and SH2 domains for the activity of asciminib in vitro, as well as sequences encoded by *ABL1* exon 2, which include the N-cap region and proximal third of the SH3 domain.

We designed a series of *BCR::ABL1* retroviral constructs that delete SH3 (“ΔSH3”), SH2 (“ΔSH2”) domains, or both (“ΔSH3/ΔSH2”). We also created constructs encoding *BCR::ABL1/b3a3*, and *BCR::ABL1/b3a2* (hereafter termed “*BCR::ABL1*”) that served as a control. All isoforms readily transformed transduced Ba/F3 cells to growth factor independence, suggesting no substantial deleterious impact upon kinase activity. As expected, all isoforms displayed equivalent sensitivity to orthosteric TKIs imatinib and dasatinib. However, relative to *BCR::ABL1*, the four isoforms harboring various deletions conferred substantial resistance to asciminib, with BCR::ABL1 ΔSH3 and BCR::ABL1/b3a3 conferring the highest degrees of resistance (EC_50_ > 10 uM) (Fig. 1B). Western immunoblot analysis confirmed that the asciminib resistance of BCR::ABL1/b3a3 occurs at the biochemical level (Fig. 1C). In alignment with these in vitro results, a CML patient with *BCR::ABL1*/b2a3 who had a modest molecular response with imatinib displayed rapid loss of response upon switching to asciminib. Response was recaptured upon reinstitution of imatinib (Fig. 1D). Kinase domain sequencing revealed no mutations.

**Fig. 2:**
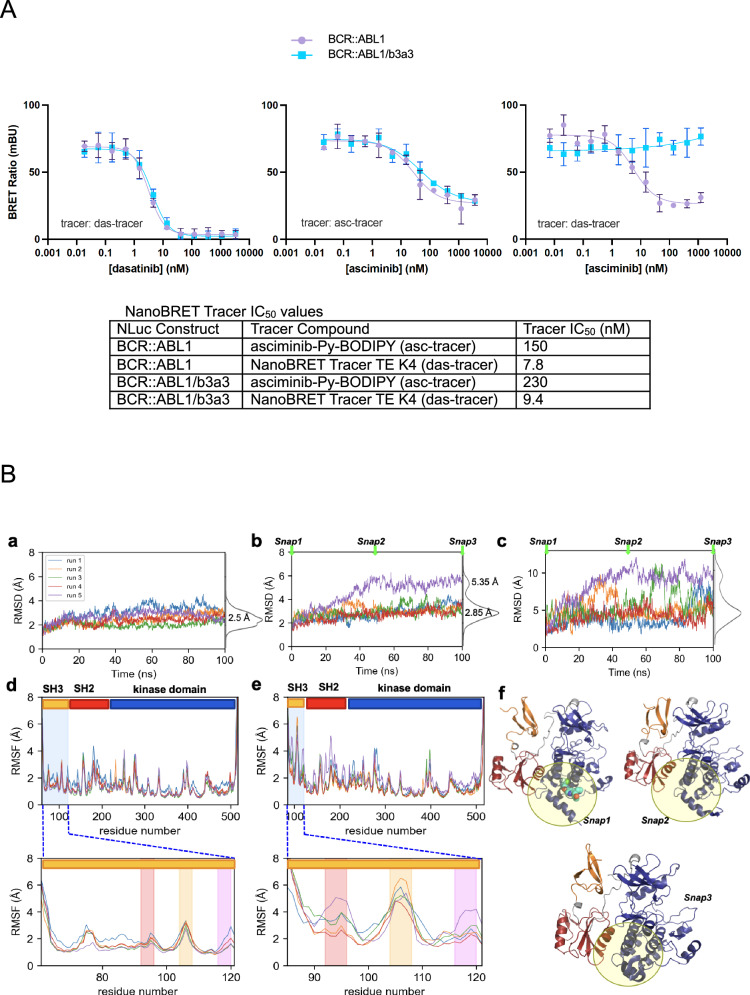
Impact of ABL1 exon 2 deletion on BCR::ABL1 TKI affinity and the stability of the ABL1 closed conformation. **A** Absence of ABL1 exon 2-encoded sequences in BCR::ABL1 decouples asciminib binding from allosteric regulation of the orthosteric site. (*Left*) dasatinib and (*Center*) asciminib bind both constructs with equal potency. (*Right*) asciminib is unable to displace the das-tracer in the context of BCR::ABL1/b3a3. Mean values and standard deviations are depicted. Experiments performed with the asc-tracer were conducted at 4x the IC_50_ of the tracer for each construct; experiments with the das-tracer were performed at 2x the IC_50_ for each construct. Table provides calculated IC_50_ values. Results were performed in technical triplicate. **B** Comparison of the dynamics of ABL1 in the presence and absence of exon 2. **a**, **b** Time evolution of the overall structure RMSD from the initial structure is shown for five MD runs of 100 ns each, conducted for (**a**) ABL1 structure composed of the kinase, SH2, and SH3 domains, and (**b**) structural model for the ABL1 Δexon 2. The density plot of the RMSDs is shown along the *right ordinate* in both cases. **c** The time evolution of the SH3 domain RMSD in the same runs as in (**b**). Careful examination shows that the fluctuations in RMSD observed in (**b**) originate from those occurring at the SH3 domain, shown in (**c**). **d**, **e** Residue RMSFs observed in the five runs shown in the respective (**a**, **b**). SH3 domain is shadowed in *blue*. *Lower panels* provide a closeup view of the change in residue dynamics within the SH3 domain. Three regions exhibiting high fluctuations in ABL1 Δexon 2 are highlighted: G92- N96 (*red*), T104-Q108 (*orange*), and I116-N120 (*magenta*), using the canonical ABL1 sequence residue numbers. **f** Three conformers (snapshots 1, 2, and 3) sampled by ABL1-Δexon2 at 0, 50, and 100 ns in *run 5* (see **b**). The SH3, SH2, and kinase domains are colored *orange*, *bordeaux*, and *dark blue*, respectively. Asciminib (not included in simulations) is displayed in snapshot 1 by *cyan* spheres so as to indicate its binding site, and the spatial neighborhood of the asciminib binding site is highlighted by semi-transparent *yellow circles* in all three snapshots. The linker connecting SH2 and kinase domains as well as that between SH2 and SH3 are colored *gray*.

To formally test whether impaired asciminib binding may contribute to resistance associated with BCR::ABL1/b3a3, NanoBRET tracer compounds (asc-tracer or das-tracer) were used to measure binding of asciminib and dasatinib to BCR::ABL1 and BCR::ABL1/b3a3 NLuc fusion proteins. The affinities of asciminib and dasatinib were comparable between the two constructs when queried using the analogous tracer compound matched for each drug. Notably, BCR::ABL1/b3a3 has similar affinity for asciminib relative to BCR::ABL1. While asciminib binding reduced the on-target occupancy of the das-tracer with low nanomolar potency in BCR::ABL1, asciminib was unable to reduce the on-target occupancy of the das-tracer in BCR::ABL1/b3a3 (Fig. 2A). Asciminib binding to BCR::ABL1 induces the autoinhibited, assembled conformation which is incompatible with dasatinib binding (Fig. 1A).

To evaluate the effect of ABL1 exon 2 (SH3 domain residues E27-K84) deletion on the stability and dynamics of ABL1 closed conformation, we generated structural models using AlphaFold2 for full-length ABL1 as well as ABL1 with exon 2 deleted (“ABL1-Δexon2”). Comparison of full-length model to the structures experimentally resolved for ABL1 kinase in the closed conformation in the presence of asciminib and an orthosteric inhibitor, SKI (PDB: 8SSN), or nilotinib (PDB: 5MO4) [2, 13] yielded root-mean-square deviations (RMSDs) of 1.29 Å and 0.49 Å, respectively, supporting the validity of the AlphaFold2 model. Both models were subjected to energy minimization and molecular dynamics (MD) simulations, which revealed significant alterations in the conformational state of ABL1-Δexon2. We removed the CAP from the full-length ABL1 to eliminate results due to the confounding presence of the CAP. We conducted five independent MD runs of 100 ns each, for both ABL1 and ABL1-Δexon2. Figure 2B panels **a**, **b** shows the time evolution of the RMSD from the original conformation for the two models. The five trajectories for ABL1 (panel **a**) consistently converged to an RMSD of approximately 3 Å within the first 20 ns, and the structure remained stable during the rest of simulations. In contrast, a greater span of RMSDs was observed in ABL1-Δexon2 (panel **b**). One of the MD runs (*run 5*) exhibited up to 6 Å RMSD. The distribution of RMSDs, plotted along the *right ordinate*, showed that two peaks centered at 2.85 Å and 5.35 Å in ABL1-Δexon2, compared to a peak at 2.5 Å in ABL1.

A closer look demonstrated that the ABL1-Δexon2 RMSD profile essentially originated from the conformational disorder in the SH3 domain that lacks E27-K84 (see Fig. 2B panel **c**). This partially truncated SH3 domain reached >10 Å RMSD within 40 ns in *run 5* (*violet curve*); and occasionally exhibited up to 8–10 Å fluctuations in *runs 1, 2 and 3* (*blue*, *orange, green*, respectively), indicative of a significant reduction in its conformational stability. The SH3 RMSD at t = 100 ns varied between 5 Å and 10 Å in all runs (panel **c**).

Next, we set out to identify the specific contributions of individual residues to the global reduced stability of ABL1-Δexon2. Figure 2B panels **d**, **e** display the root-mean-square fluctuations (RMSFs) of residues observed for ABL1 and ABL1-Δexon2, respectively. As expected, SH3 domain residues (*shaded in blue*) experienced the most elevated fluctuations upon the removal of exon 2, followed by the SH2 domain. A closer look at the non-exon2 portion of the SH3 domain (insets of Fig. 2B panels **d**, **e**) revealed three regions distinguished by enhanced fluctuations: G92-N96 (*red*) on the loop connecting the first two β-strands; T104-Q108 (*orange*), between the second and third β-strands; and I116-N120 (*magenta*) on the link connecting SH3 and SH2 domains. T104-Q108 underwent more than 4 Å displacements in all runs conducted for ABL1-Δexon2. Three snapshots (*Snaps 1, 2, and 3*) from *run 5* at 0, 50, and 100 ns (Fig. 2B panel **f**) illustrate the conformational changes originating from exon 2 deletion: the SH3 β-sheet (*orange*) changes its orientation from horizontal (*snap1*) to vertical (*snap2*) in conjunction with the dissociation of the first strand from the β-sheet. The first strand further departs and adopts a direction perpendicular to the other two at 100 ns. Notably, the SH2 domain (*colored bordeaux*) undergoes a global rotation, which directly affects its interaction with the asciminib-binding (myristoylation) site. *Snap 1* displays bound asciminib (van der Waals representation; *cyan*), to indicate the binding site of asciminib; and the *semi-transparent yellow circle*s on the three snapshots highlight the structural changes occurring during the course of simulations in the close vicinity of this binding site. The observed change in the packing of SH2 against the kinase C-lobe would interfere with the action of asciminib.

Overall, these simulations demonstrate that the stability of ABL1 is impacted by the loss of exon 2. Deletion of the segment E27-K84 induces an enhanced mobility and loss of structure in the SH3 domain, and significantly, the SH2 domain itself undergoes an overall reorientation accompanying the disorder and fluctuations in the conformation of the SH3 domain. The enhanced fluctuations of SH3 and SH2 residues, the gain of SH3 mobility and the alterations in SH3 and SH2 conformations, all associated with the deletion of exon 2, provide potential avenues to deviate from the asciminib-bound closed conformation, and to interfere with the allosteric effect of asciminib.

Here we demonstrate the critical importance of the SH3 and SH2 domains for the kinase inhibitor activity of asciminib. Moreover, sequences encoded by ABL1 exon 2 are similarly essential for the ability of asciminib to induce a closed, inactive kinase conformation. Computational studies suggest deletion of the residues encoded by exon 2 in the SH3 domain not only impacts the conformation of the SH3 domain, but also that of the SH2 domain and thereby inter-domain interactions near the asciminib-binding site. While clinical experience of asciminib in CML patients with BCR::ABL1/b3a3 is extremely limited, based on the data presented here and by others [14, 15], asciminib should only be used with extreme caution under close molecular monitoring in this patient population. Given the high degree of asciminib resistance observed despite no significant impact upon the ability of asciminib to bind to BCR::ABL1/b3a3, it appears highly likely that emerging TKIs that target the myristoyl-binding pocket will be similarly ineffective in patients with this variant. Our findings further raise the possibility that acquired asciminib resistance could arise through mutation of the ABL1-exon2 splice acceptor site causing skipping. Translational studies of appropriate samples will be necessary to test these hypotheses.

### Supplementary information

Supplemental Information
