# Supplementary material for: Absence of ABL1 exon 2-encoded SH3 residues in BCR::ABL1 destabilizes the autoinhibited kinase conformation and confers resistance to asciminib
Source: Leukemia. 2024 Jul 31;38(9):2046–50. doi: 10.1038/s41375-024-02353-0 (PMC11347358; doi:10.1038/s41375-024-02353-0)
Supplement: Supplementary file 1 — Supplemental Information [file 41375_2024_2353_MOESM1_ESM.docx]

**Supplemental Information**

**Absence of *ABL1* exon 2-encoded SH3 residues in *BCR::ABL1* destabilizes the autoinhibited kinase conformation and confers resistance to asciminib**

Ariel Leyte-Vidal^1,2^, RosaAnna DeFilippis^1^, Ian R. Outhwaite^3^, Isabelle Kwan^3,4,5^, Ji Young Lee^4^, Carlyn Leavitt^1^, Kaeli B. Miller^1^, Delphine Rea^6^, Aziz M. Rangwala^3^, Kevin Lou^7,8^, Suhana Patel^1^, Ailin Alvarez^1^, Kevan M. Shokat^7,8,9^, Ivet Bahar^4,5^, Markus A. Seeliger^3^ and Neil P. Shah^1^

^1^Division of Hematology/Oncology, Department of Medicine, University of California, San Francisco, San Francisco CA 94143, USA

^2^Department of Biochemistry and Molecular Biology, University of Miami Miller School of Medicine, Miami, FL 33101, USA

^3^Department of Pharmacological Sciences, Renaissance School of Medicine, Stony Brook University, Stony Brook, NY 11794, USA

^4^Laufer Center for Physical and Quantitative Biology, Stony Brook University, Stony Brook, NY 11794, USA

^5^Department of Biochemistry and Cell Biology, Renaissance School of Medicine, Stony Brook University, Stony Brook, NY 11794, USA

^6^Adult Hematology Department, Hôpital Saint-Louis, Paris, France

^7^Department of Cellular and Molecular Pharmacology, University of California, San Francisco, San Francisco, CA 94158, USA

^8^Howard Hughes Medical Institute, University of California, San Francisco, San Francisco, CA 94158, USA

^9^Department of Chemistry, University of California, Berkeley, Berkeley, CA 94720, USA.

Corresponding author:

Neil P. Shah

Division of Hematology/Oncology, Department of Medicine, University of California, San Francisco, San Francisco CA 94143, USA

Email: [neil.shah@ucsf.edu](mailto:neil.shah@ucsf.edu)

**Content**

**Supplementary Materials and Methods 3**

**References 10**

**Supplementary Materials and Methods**

*DNA construct design and creation*

To create MSCVpuroBCR::ABL1 with various deletions, gBlocks (Integrated DNA Technologies; itdna.com) were designed to span the region of BCR::ABL b3a2 between the HindIII and BsrGI restriction enzyme sites that contains ABL1 exon 2 and sequences encoding the SH3 and SH2 domains. The resultant gBlock sequences are shown below:

**ΔSH3:**

5’aagcttctccctgacatccgtggagctgcagatgctgaccaactcgtgtgtgaaactccagactgtccacagcattccgctgaccatcaataaggaagatgatgagtctccggggctctatgggtttctgaatgtcatcgtccactcagccactggatttaagcagagttcaaaagcccttcagcggccagtagcatctgactttgagcctcagggtctgagtgaagccgctcgttggaactccaaggaaaaccttctcgctggacccagtgaaaatgaccccaacctggagaaacactcctggtaccatgggcctgtgtcccgcaatgccgctgagtatctgctgagcagcgggatcaatggcagcttcttggtgcgtgagagtgagagcagtcctggccagaggtccatctcgctgagatacgaagggagggtgtaccattacaggatcaacactgcttctgatggcaagctctacgtctcctccgagagccgcttcaacaccctggccgagttggttcatcatcattcaacggtggccgacgggctcatcaccacgctccattatccagccccaaagcgcaacaagcccactgtctatggtgtgtcccccaactacgacaagtgggagatggaacgcacggacatcaccatgaagcacaagctgggcgggggccagtacggggaggtgtacgagggcgtgtggaagaaatacagcctgacggtggccgtgaagaccttgaaggaggacaccatggaggtggaagagttcttgaaagaagctgcagtcatgaaagagatcaaacaccctaacctggtgcagctccttggggtctgcacccgggagcccccgttctatatcatcactgagttcatgacctacgggaacctcctggactacctgagggagtgcaaccggcaggaggtgaacgccgtggtgctgctgtaca-3’

**ΔSH2**

5’aagcttctccctgacatccgtggagctgcagatgctgaccaactcgtgtgtgaaactccagactgtccacagcattccgctgaccatcaataaggaagatgatgagtctccggggctctatgggtttctgaatgtcatcgtccactcagccactggatttaagcagagttcaaaagcccttcagcggccagtagcatctgactttgagcctcagggtctgagtgaagccgctcgttggaactccaaggaaaaccttctcgctggacccagtgaaaatgaccccaaccttttcgttgcactgtatgattttgtggccagtggagataacactctaagcataactaaaggtgaaaagctccgggtcttaggctataatcacaatggggaatggtgtgaagcccaaaccaaaaatggccaaggctgggtcccaagcaactacatcacgccagtcaacagtaacaagcccactgtctatggtgtgtcccccaactacgacaagtgggagatggaacgcacggacatcaccatgaagcacaagctgggcgggggccagtacggggaggtgtacgagggcgtgtggaagaaatacagcctgacggtggccgtgaagaccttgaaggaggacaccatggaggtggaagagttcttgaaagaagctgcagtcatgaaagagatcaaacaccctaacctggtgcagctccttggggtctgcacccgggagcccccgttctatatcatcactgagttcatgacctacgggaacctcctggactacctgagggagtgcaaccggcaggaggtgaacgccgtggtgctgctgtaca-3’

**ΔSH3/ΔSH2**

5’aagcttctccctgacatccgtggagctgcagatgctgaccaactcgtgtgtgaaactccagactgtccacagcattccgctgaccatcaataaggaagatgatgagtctccggggctctatgggtttctgaatgtcatcgtccactcagccactggatttaagcagagttcaaaagcccttcagcggccagtagcatctgactttgagcctcagggtctgagtgaagccgctcgttggaactccaaggaaaaccttctcgctggacccagtgaaaatgaccccaacaacaagcccactgtctatggtgtgtcccccaactacgacaagtgggagatggaacgcacggacatcaccatgaagcacaagctgggcgggggccagtacggggaggtgtacgagggcgtgtggaagaaatacagcctgacggtggccgtgaagaccttgaaggaggacaccatggaggtggaagagttcttgaaagaagctgcagtcatgaaagagatcaaacaccctaacctggtgcagctccttggggtctgcacccgggagcccccgttctatatcatcactgagttcatgacctacgggaacctcctggactacctgagggagtgcaaccggcaggaggtgaacgccgtggtgctgctgtaca-3’

**b3a3**

5’aagcttctccctgacatccgtggagctgcagatgctgaccaactcgtgtgtgaaactccagactgtccacagcattccgctgaccatcaataaggaagatgatgagtctccggggctctatgggtttctgaatgtcatcgtccactcagccactggatttaagcagagttcaagtgaaaagctccgggtcttaggctataatcacaatggggaatggtgtgaagcccaaaccaaaaatggccaaggctgggtcccaagcaactacatcacgccagtcaacagtctggagaaacactcctggtaccatgggcctgtgtcccgcaatgccgctgagtatctgctgagcagcgggatcaatggcagcttcttggtgcgtgagagtgagagcagtcctggccagaggtccatctcgctgagatacgaagggagggtgtaccattacaggatcaacactgcttctgatggcaagctctacgtctcctccgagagccgcttcaacaccctggccgagttggttcatcatcattcaacggtggccgacgggctcatcaccacgctccattatccagccccaaagcgcaacaagcccactgtctatggtgtgtcccccaactacgacaagtgggagatggaacgcacggacatcaccatgaagcacaagctgggcgggggccagtacggggaggtgtacgagggcgtgtggaagaaatacagcctgacggtggccgtgaagaccttgaaggaggacaccatggaggtggaagagttcttgaaagaagctgcagtcatgaaagagatcaaacaccctaacctggtgcagctccttggggtctgcacccgggagcccccgttctatatcatcactgagttcatgacctacgggaacctcctggactacctgagggagtgcaaccggcaggaggtgaacgccgtggtgctgctgtaca-3’

gBlocks were first ligated into pBluescriptII KS+ cut with EcoRV and validated by sequencing. The BsrGI/HindIII inserts were excised and ligated to pBluescript BCR::ABL1 in a 3-way manner. Specifically, pBluescript BCR::ABL1 was digested with BsrGI and ClaI, and a 3184 bp fragment was isolated. Separately, pBluescript BCR::ABL1 was digested with ClaI and HindIII and a 5645 bp fragment was isolated. These two fragments constituted the vector for ligation to the HindIII/BsrGI inserts. Subsequently, an approximately 6 kb SalI/EcoRI fragment that extends from the first exon of the BCR gene through the end of the ABL1 untranslated region was isolated for each deletion. This fragment was then ligated in a 3-way manner as follows. MSCVpuroBCR::ABL1 was digested with EcoR1/ClaI and a 1.2 kb fragment was isolated. Separately, MSCVpuroBCR::ABL1 was digested with ClaI/SalI and a 5.9 kb fragment was isolated. All final constructs were sequenced in their entirety.

To create the nLUC-fusion constructs used in the nanoBRET assay, a 4.3 kb XhoI/SrfI fragment extending from the first exon of BCR to ABL1 to beyond the region encoded by the kinase domain was isolated from MSCVpuroBCR::ABL1/b3a3 and ligated to a 6.4 kb fragment XhoI/SrfI fragment from pBCR::ABL1 Nn5K2 nBRET. Final constructs were sequenced in their entirety.

*Generation of Ba/F3 cell lines and EC_50_ determination*

MSCVpuroBCR::ABL1 and the four deletion constructs were cotransfected with EcoPak into 293T cells and the subsequent virus was used to infect and select Ba/F3 cell populations as previously described^1^. Following retroviral transduction, 10% FBS + RPMI and il3 were added and incubated for 18 hrs. Cells were washed three times to remove virus and then puro selected. After puro selection, growth factor IL3 was removed. Growth factor independent pools were treated in technical triplicate with DMSO (vehicle control), imatinib, dasatinib, or asciminib at escalating concentrations. After 48 hours, cellular proliferation was assessed by CellTiter-Glo. Three biological replicates were performed. EC_50_ values were determined using Prism software.

*Western immunoblot analysis*

Western immunoblot: biochemical assessments of TKI inhibition in Ba/F3 cellular extracts were performed following two hours of TKI treatment as previously described^1^. Eight million cells were treated in 8 mLs of media at a concentration of 1x10^6 cells/mL per condition with (0, 1, 10, 100, 1000, 10,000 nM) drug for two hours. Cells were then spun down, washed with PBS, and pellets were resuspended in NP40 lysis buffer containing protease and phosphatase inhibitors. Protein lysates were normalized to a concentration of 2ug/uL. Forty uL of protein lysates per condition were loaded onto an Invitrogen NuPage 4-12% Bis-Tris Midi Gel. The following antibodies were used: anti-ABL1 (phospho tyr412) (Abcam), anti-ABL1 (BD Biosciences), pSTAT5, STAT5, GAPDH (Cell Signaling). Imaging was performed on the BIORAD Chemidoc MP Imaging System.

*Patient information*

The CML patient highlighted in Fig 1D was diagnosed with Philadelphia chromosome-positive, BCR::ABL1/b2a3 variant chronic phase CML, Sokal/EUTOS/ELTS low score, in 2013. The patient initiated imatinib 400 mg daily and achieved a complete cytogenetic response after six months of therapy. The patent was briefly switched to dasatinib 100 mg daily after one year in an effort to achieve a deeper molecular response and improved quality of life, but developed dermatotoxicity and returned to imatinib 400 mg daily. While the molecular response with imatinib was relatively stable, cytogenetic relapse was noted after 3.5 years, when 1/20 bone marrow metaphases displayed the Philadelphia chromosome. No TKI-resistant mutations were identified. The patient was then treated with a combination of imatinib 400 mg and asciminib 60 mg daily for approximately three months, at which time imatinib was stopped and asciminib 60 mg was continued. After eight weeks, the transcript level rose from 0.57 to 15, and subsequently to 55 one month later on asciminib monotherapy. Two weeks later, imatinib 300 mg was reintroduced with continuation of asciminib 60 mg.  The transcript level declined to 4.2 after one month, and asciminib was then discontinued while imatinib was escalated to 400 mg daily.  The patient has maintained a stable molecular response on imatinib 400 mg daily for more than five years. No kinase domain mutations have been detected in this case.

*BCR::ABL1/b2a3 transcript quantification in clinical case*

Forward primers specific for *BCR* exon 13 were used in combination with TaqMan probe ENPr1043 and reverse primer ENR1063 for fusion involving *ABL1* exon 3. *ABL1* was used as a control gene to normalize *BCR::ABL1* transcript expression levels in a non-IS fashion^2^. *BCR::ABL1* ratios were calculated using the delta Ct method, after checking that PCR amplification efficiency measured for the fusion and control genes were optimal^3^.

*NanoBRET assay*

NanoLuc constructs (full-length BCR::ABL1, full-length BCR::ABL1/b3a3) were transfected into HEK293T cells using FuGENE HD (Promega) and plated at 2x10^5^ cells/mL in DMEM (Corning) without fetal bovine serum supplementation into 384-well plates (Corning). Cells were incubated at 37°C and 5% CO_2_ for 24 hours. Serially diluted unlabeled drugs, tracer compounds, and/or DMSO controls were added to cells. Experiments with NanoBRET TE Tracer K4 (das-tracer, Promega) were conducted at 2X the IC50 of the tracer for each construct, and experiments with asciminib-Py-BODIPY (asc-tracer) were conducted at 4X the IC50 of the tracer for each construct due to the low assay window. Following addition of tracer compounds and unlabeled drugs the cells were equilibrated at 37°C and 5% CO_2_ for 2 hours. BRET signal was read following application of NanoBRET NanoGlo Substrate (Promega) and Extracellular NanoLuc Inhibitor (Promega) using a Synergy Neo2 plate reader (Agilent BioTek). The average BRET ratio (610nm/450nm) in miliBRET units (mBU) of the no-tracer background controls for each condition were subtracted from the BRET ratio in mBU of the associated wells, and the data was fit in GraphPad Prism 10 using a four-parameter ([Inhibitor] vs. response) model.

The asciminib-based NanoBRET target engagement tracer, asciminib-Py-BODIPY retains the N-(4-(chlorodifluoromethoxy)phenyl)-5-(1H-pyrazol-5-yl)nicotinamide core in asciminib responsible for engaging the myristoyl pocket^4^ and substitutes the (*R*)-3-pyrrolidinol moiety with a similar piperidine group that enables linkage to a fluorescent energy transfer group. We anticipated that this chemical modification would have minimal effect on binding interactions with the myristoyl pocket since the position is solvent-exposed^4^ and tolerates a variety of different chemical substitutions^5^. Py-BODIPY was selected as a fluorophore that is compatible with previously reported NanoBRET donor-acceptor kinase target engagement systems^6,7^.

*Chemical synthesis of asciminib-Py-BODIPY*

Nuclear magnetic resonance (NMR) spectra were recorded on a Bruker spectrometer at 400 MHz. Chemical shifts were reported as parts per million (ppm) from solvent references. Liquid chromatography-mass spectrometry (LC-MS) was performed on a Waters Xevo G2-XS QTof (0.6 mL/min) using an ACQUITY UPLC BEH C18 column (Waters) and a water-acetonitrile gradient (0.05% formic acid) using Optima LC-MS grade solvents (Fisher Scientific). All other solvents (Fisher Scientific, Millipore Sigma) and commercially available reagents were used without further purification. Analytical thin-layer chromatography was performed with silica gel 60 F254 glass plates (Millipore Sigma). Flash chromatography was performed with RediSep Rf normal-phase silica flash columns using a CombiFlash Rf+ (Teledyne ISCO). Preparative high-performance liquid chromatography (HPLC) was performed on a CombiFlash EZ Prep using a RediSep C18 Prep HPLC Column (Teledyne ISCO) with a water-acetonitrile gradient (0.1% formic acid).

**Reagents and conditions**. (a) HATU, DIPEA, DMF, rt, 98%. (b) TFA, CH_2_Cl_2_, rt. (c) HATU, DIPEA, DMF, rt, 38% over two steps. Abbreviations: HATU, 1-[bis(dimethylamino)methylene]-1*H*-1,2,3-triazolo[4,5-b]pyridinium 3-oxide hexafluorophosphate; DIPEA, *N*,*N*-diisopropylethylamine; DMF, *N*,*N*-dimethylformamide; TFA, trifluoroacetic acid.

**Compound 1** was obtained by custom synthesis (WuXi AppTec) employing methods similar to those reported previously^8^.

**Compound 2**. To a mixture of compound 1 (30 mg, 0.0521 mmol) and *t*-Boc-*N*-amido-PEG2-amine (14 mg, 0.0573 mmol) in *N*,*N*-dimethylformamide (0.521 mL) was added *N*,*N*-diisopropylethylamine (27 μL, 0.156 mmol). The solution was cooled in an ice-water bath before the addition of 1-[bis(dimethylamino)methylene]-1*H*-1,2,3-triazolo[4,5-b]pyridinium 3-oxide hexafluorophosphate (22 mg, 0.0573 mmol) and stirred at room temperature overnight. The mixture was partitioned between ethyl acetate and saturated sodium bicarbonate and the organic layer was washed with saturated sodium bicarbonate (2×), water (4×), brine (2×), dried over sodium sulfate, filtered, and concentrated *in vacuo*. The crude was purified by flash chromatography over silica gel eluting with a gradient from 0% methanol-dichloromethane to 10% methanol-dichloromethane to afford compound 2 (41 mg, 0.0508 mmol, 98% yield) as a white solid.

**^1^H NMR** (400 MHz, DMSO-*d*_6_) δ 10.33 (br. s, 1H), 8.89 – 8.78 (m, 1H), 8.14 – 8.01 (m, 1H), 7.86 (d, *J* = 9.1 Hz, 2H), 7.83 – 7.77 (m, 1H), 7.70 – 7.60 (m, 1H), 7.35 (d, *J* = 8.7 Hz, 2H), 6.81 – 6.68 (m, 1H), 6.51 – 6.42 (m, 1H), 5.23 – 5.10 (m, 1H), 3.87 – 3.75 (m, 1H), 3.75 – 3.59 (m, 2H), 3.54 – 3.42 (m, 4H), 3.39 – 3.34 (m, 4H), 3.22 – 3.11 (m, 2H), 3.10 – 2.99 (m, 2H), 2.82 – 2.62 (m, 2H), 2.42 – 2.23 (m, 2H), 2.05 – 1.81 (m, 2H), 1.64 – 1.39 (m, 8H), 1.36 (s, 9H).

**^19^F NMR** (376 MHz, DMSO-*d*_6_) δ -24.7.

**HRMS** (*m/z*): calculated for C_38_H_51_ClF_2_N_7_O_8_^+^ [M + H]^+^ 806.3450, found 806.3438.

**TLC**: R*_f_* = 0.5 (10% methanol-dichloromethane, UV).

**Asciminib-Py-BODIPY**. To a mixture of compound 2 (34 mg, 0.0422 mmol) in dichloromethane (0.422 mL) was added trifluoroacetic acid (0.422 mL). The solution was stirred at room temperature for 6 h before concentrating *in vacuo* to afford an amber oil that was used directly in the next step. To a mixture of the crude amine, trifluoroacetic acid salt and Py-BODIPY-acid (14 mg, 0.0422 mmol) in *N*,*N*-dimethylformamide (0.422 mL) was added *N*,*N*-diisopropylethylamine (73 μL, 0.422 mmol). The solution was cooled in an ice-water bath before the addition of 1-[bis(dimethylamino)methylene]-1*H*-1,2,3-triazolo[4,5-b]pyridinium 3-oxide hexafluorophosphate (16 mg, 0.0422 mmol) and stirred at room temperature overnight. The mixture was partitioned between ethyl acetate and saturated sodium bicarbonate and the organic layer was washed with saturated sodium bicarbonate (2×), water (4×), brine (2×), dried over sodium sulfate, filtered, and concentrated *in vacuo*. The crude was purified by HPLC to afford asciminib-Py-BODIPY (15 mg, 0.0161 mmol, 38% yield over two steps) as a blue solid.

**^1^H NMR** (400 MHz, DMSO-*d*_6_) δ 13.02 (br. s, 1H), 11.41 (br. s, 1H), 10.40 (br. s, 1H), 8.78 – 8.70 (m, 1H), 8.33 (br. s, 1H), 8.06 – 7.98 (m, 1H), 7.96 – 7.74 (m, 4H), 7.43 (s, 1H), 7.38 – 7.30 (m, 4H), 7.29 – 7.25 (m, 1H), 7.16 (d, *J* = 4.6 Hz, 1H), 7.01 (d, *J* = 4.0 Hz, 1H), 6.65 (br. s, 1H), 6.37 – 6.30 (m, 2H), 3.74 – 3.55 (m, 2H), 3.54 – 3.47 (m, 4H), 3.45 – 3.36 (m, 4H), 3.26 – 3.16 (m, 4H), 3.14 (t, *J* = 7.7 Hz, 2H), 2.79 – 2.61 (m, 2H), 2.54 – 2.53 (m, 2H), 2.35 – 2.22 (m, 1H), 1.71 – 1.50 (m, 4H).

**^19^F NMR** (376 MHz, DMSO-*d*_6_) δ -24.7 (2F), -142.3 (m, 2F).

**HRMS** (*m/z*): calculated for C_44_H_47_BClF_4_N_10_O_6_^+^ [M + H]^+^ 933.3392, found 933.3429.

*Molecular dynamics simulation*

We modeled the ABL1 and ABL1 Δexon 2 structures using AlphaFold2^9^. All-atom MD systems for the modeled structures were set up using CHARMM-GUI solution builder^10^, and simulations were performed using NAMD^11^ with the CHARMM36m force field^12^ for protein and the TIP3P water model^13^. We performed five independent runs of 100 ns for each system (total 0.5 ms each). We relaxed the systems using the equilibration steps in CHARMM-GUI and performed NPT dynamics with a 2 fs time step. Nosé-Hoover constant pressure (1 bar) and temperature (310 K) were used. RMSD and RMSF were analyzed using VMD 1.9.4^14^. Structure visualization was performed using the PyMOL Molecular Graphics, Version 2.3.5, Schrödinger, LLC.

REFERENCES

1. Shah NP, Nicoll JM, Nagar B, et al. Multiple BCR-ABL kinase domain mutations confer polyclonal resistance to the tyrosine kinase inhibitor imatinib (STI571) in chronic phase and blast crisis chronic myeloid leukemia. *Cancer Cell.* 2002;2(2):117-125.

2. Beillard E, Pallisgaard N, van der Velden VH, et al. Evaluation of candidate control genes for diagnosis and residual disease detection in leukemic patients using 'real-time' quantitative reverse-transcriptase polymerase chain reaction (RQ-PCR) - a Europe against cancer program. *Leukemia.* 2003;17(12):2474-2486.

3. Gabert J, Beillard E, van der Velden VH, et al. Standardization and quality control studies of 'real-time' quantitative reverse transcriptase polymerase chain reaction of fusion gene transcripts for residual disease detection in leukemia - a Europe Against Cancer program. *Leukemia.* 2003;17(12):2318-2357.

4. Wylie AA, Schoepfer J, Jahnke W, et al. The allosteric inhibitor ABL001 enables dual targeting of BCR-ABL1. *Nature.* 2017;543(7647):733-737.

5. Schoepfer J, Jahnke W, Berellini G, et al. Discovery of Asciminib (ABL001), an Allosteric Inhibitor of the Tyrosine Kinase Activity of BCR-ABL1. *J Med Chem.* 2018;61(18):8120-8135.

6. Vasta JD, Corona CR, Wilkinson J, et al. Quantitative, Wide-Spectrum Kinase Profiling in Live Cells for Assessing the Effect of Cellular ATP on Target Engagement. *Cell Chem Biol.* 2018;25(2):206-214 e211.

7. Lyczek A, Berger BT, Rangwala AM, et al. Mutation in Abl kinase with altered drug-binding kinetics indicates a novel mechanism of imatinib resistance. *Proc Natl Acad Sci U S A.* 2021;118(46).

8. Lou K, Wassarman DR, Yang T, et al. IFITM proteins assist cellular uptake of diverse linked chemotypes. *Science.* 2022;378(6624):1097-1104.

9. Jumper J, Evans R, Pritzel A, et al. Highly accurate protein structure prediction with AlphaFold. *Nature.* 2021;596(7873):583-589.

10. Jo S, Kim T, Iyer VG, Im W. CHARMM-GUI: a web-based graphical user interface for CHARMM. *J Comput Chem.* 2008;29(11):1859-1865.

11. Phillips JC, Braun R, Wang W, et al. Scalable molecular dynamics with NAMD. *J Comput Chem.* 2005;26(16):1781-1802.

12. Huang J, Rauscher S, Nawrocki G, et al. CHARMM36m: an improved force field for folded and intrinsically disordered proteins. *Nat Methods.* 2017;14(1):71-73.

13. Jorgensen WL, Chandrasekhar, J., Madura, J. D., Impey, R. W., Klein, M. L. . Comparison of simple potential functions for simulating liquid water. *The Journal of Chemical Physics.* 1983;79:926-935.

14. Humphrey W, Dalke A, Schulten K. VMD: visual molecular dynamics. *J Mol Graph.* 1996;14(1):33-38, 27-38.
